# A pandemic strain of calicivirus threatens rabbit industries in the Americas

**DOI:** 10.1186/1743-422X-4-96

**Published:** 2007-10-02

**Authors:** Michael T McIntosh, Shawn C Behan, Fawzi M Mohamed, Zhiqiang Lu, Karen E Moran, Thomas G Burrage, John G Neilan, Gordon B Ward, Giuliana Botti, Lorenzo Capucci, Samia A Metwally

**Affiliations:** 1 Foreign Animal Disease Diagnostic Laboratory, Animal and Plant Health Inspection Services, United States Department of Agriculture, Plum Island Animal Disease Center, P.O. Box 848, Greenport, NY 11944, USA; 2 Department of Homeland Security, Plum Island Animal Disease Center, P.O. Box 848, Greenport, NY 11944, USA; 3 Istituto Zooprofilattico Sperimentale della Lombardia ed Emilia Romagna via Bianchi, 9 – 25124 Brescia, Italy

## Abstract

Rabbit Hemorrhagic Disease (RHD) is a severe acute viral disease specifically affecting the European rabbit *Oryctolagus cuniculus*. As the European rabbit is the predominant species of domestic rabbit throughout the world, RHD contributes towards significant losses to rabbit farming industries and endangers wild populations of rabbits in Europe and other predatory animals in Europe that depend upon rabbits as a food source. Rabbit Hemorrhagic Disease virus (RHDV) – a *Lagovirus *belonging to the family *Caliciviridae *is the etiological agent of RHD. Typically, RHD presents with sudden death in 70% to 95% of infected animals. There have been four separate incursions of RHDV in the USA, the most recent of which occurred in the state of Indiana in June of 2005. Animal inoculation studies confirmed the pathogenicity of the Indiana 2005 isolate, which caused acute death and pathological changes characterized by acute diffuse severe liver necrosis and pulmonary hemorrhages. Complete viral genome sequences of all USA outbreak isolates were determined and comparative genomics revealed that each outbreak was the result of a separate introduction of virus rather than from a single virus lineage. All of the USA isolates clustered with RHDV genomes from China, and phylogenetic analysis of the major capsid protein (VP60) revealed that they were related to a pandemic antigenic variant strain known as RHDVa. Rapid spread of the RHDVa pandemic suggests a selective advantage for this new subtype. Given its rapid spread, pathogenic nature, and potential to further evolve, possibly broadening its host range to include other genera native to the Americas, RHDVa should be regarded as a threat.

## Introduction

Rabbit Hemorrhagic Disease (RHD) is a highly contagious, severe acute viral illness that specifically afflicts rabbits of the species *Oryctolagus cuniculus*. Since its emergence in 1984, RHD has resulted in the deaths of nearly a quarter billion free-living and domestic rabbits. While RHDV is not known to affect humans or any other animal species, it continues to generate significant losses to rabbit farming industries and trade. Typically, the disease presents with fever and sudden death within the first 12 to 36 hours after natural exposure. Rabbits will often develop a blood-tinged foamy nasal discharge, severe respiratory distress and/or convulsions preceding death [1,2]. Mortality rates are high, ranging from 70% to 95%. However, 5% to 10% of infected rabbits may display an illness that presents with jaundice, malaise, weight-loss, and eventual death within 1 to 2 weeks of onset. As an exception, rabbits under 45–50 days of age survive infection without the presentation of clinical signs, although they are suspected of carrying the infection [3]. Humoral immunity is critical to protection from RHD, and an effective vaccine produced from liver homogenates of infected rabbits is employed to protect breeding rabbits in all countries where RHD is endemic [4].

The etiological agent of RHD is the Rabbit Hemorrhagic Disease Virus (RHDV), a member of the family *Caliciviridae *[5-8]. In addition to RHD, this family of viruses comprises a number of important human and animal pathogens including noroviruses or Norwalk-like viruses, which cause severe gastroenteritis in humans, and vesiviruses like the vesicular exanthema of swine virus. A similar virus, the European Brown Hare Syndrome Virus (EBHSV), afflicts the European hares of the *Lepus *genus [9]. The nearest relation to RHDV, however, is a non-pathogenic calicivirus named Rabbit Calicivirus (RCV) [10]. These three viruses of *Lagomorphs *(RHDV, RCV and EBHSV) comprise a recently formed *Lagovirus *genus within the family *Caliciviridae *[11].

RHDV like other caliciviruses forms 28–32 nm diameter, non-enveloped, icosohedral virus particles that harbor a 7.4 kb positive or sense oriented single-stranded RNA genome that encodes a 257 kDa polyprotein [12,13]. Post-translational processing at 8 proteolytic cleavage sites within this polyprotein gives rise to several mature nonstructural proteins including a helicase, protease, and RNA-dependent RNA-polymerase, as well as to the 60 kDa major capsid protein/antigen (VP60) [14-16]. This same VP60 is also known to be expressed from a downstream 2.4 kb subgenomic mRNA that arises from an alternate transcriptional start site [17,18]. An additional minor capsid protein is expressed downstream of the VP60 by virtue of a novel translational termination and reinitiating mechanism [19,20].

RHDV is environmentally stable, highly infectious, and transmissible by close contact or by contact with fomites such as contaminated fur, clothing, or cages. Indirect arthropod vectors, including blow flies or flesh flies, have also been implicated in the spread of RHDV [21]. Since its characterization from a large outbreak in 1984 that killed over 140 million rabbits in China [22], the spread of RHD throughout the world has been rapid. RHD was reported in Italy in 1986 [23], and it became endemic in Europe by 1990 [24]. In 1988, RHD was reported in Korea and Mexico; both outbreaks have been linked to the importation of rabbit products from China [25,26]. *O. cuniculus *is not native to Mexico, and in 1989 the government of Mexico initiated a successful eradication campaign. To date Mexico remains free of RHD. RHDV was inadvertently introduced into Australia by a breach in biocontainment during studies aimed at developing RHDV as a biological control agent for feral rabbit population reduction [27-29]. It spread rapidly throughout Australia, leading to its illegal introduction into New Zealand in 1997 by farmers attempting to reduce local rabbit populations [30-32]. Today, RHD is endemic in China, Korea, Europe, Morocco, Cuba, Australia, and New Zealand.

Only a single serotype of RHDV is known to exist [33,34]. Of particular interest however, has been the emergence of an antigenic variant strain or subtype of RHDV known as RHDVa [35,36]. For instance, RHDVa is replacing original strains of RHDV in Italy [37]. Likewise, two recent French isolates belonging to the RHDVa antigenic subtype have been identified [38]. Phylogenetic analysis of partial VP60 sequences from isolates dating back to 1988 also revealed the emergence of the RHDVa strain in a 2003 outbreak of RHD in Hungary [39]. Likewise, the World Organization for Animal Health (OIE) has reported that the RHDVa subtype was responsible for the first ever recorded outbreak of RHD in Uruguay near the end of November of 2004. At the same time, a large outbreak of RHD attributed to RHDVa occurred in Cuba [40]. Most recently, the RHDVa subtype was isolated from wild rabbits in the Netherlands and has been suggested as a possible reason for the recent decline in free living *O. cuniculus *rabbits in that country [41]. These combined observations confirm that the spread of RHDVa is a pandemic and suggest a selective advantage for infectivity or replication of RHDVa over the original serotype of RHDV.

The USA has experienced four sporadic incursions of RHDV, the first of which occurred in Crawford County, Iowa in April of 2000 (IA-00) [42]. In August of 2001, an outbreak of RHD was reported in Utah County, Utah (UT-01) and was traced to a shipment and subsequent outbreak in Illinois. In December of 2001 an isolated outbreak occurred at a zoo in Flushing, New York (NY-01), suspected to have resulted from the importation of rabbit meat from China. Most recently in 2005, an outbreak of RHD occurred at a rabbit farm in Vanderburgh County, Indiana (IN-05) with an epidemiological link to the purchase of animals from an open market in Kentucky. No positive cases have been reported from Kentucky, and in all U.S. outbreaks the origins of the virus remained indeterminable.

In this paper we describe the pathogenesis of the IN-05 outbreak isolate from the USA and for the first time compare the complete viral genomes of all U.S. isolates with genomes of other RHDV isolates throughout the world.

## Results

### U.S. RHDV Genomic Sequences

Complete genomes for the four U.S. RHDV isolates (IA-00, UT-01, NY-01, and IN-05) were determined by direct sequencing of overlapping RT-PCR products and by direct sequencing of 5' and 3' RACE products. For comparison, the full genome sequence of an Italy isolate (Italy 90) and partial sequence of a Korean isolate (Korea 90) lacking only the extreme 5' end were determined. Like the NCBI reference RHDV genome (acc#: NC_001543), each of the four U.S. RHDV genomes had a length of 7,437 nt and had an additional poly A tail of undetermined length. The U.S. isolates shared 89–90% nucleotide sequence identity with the viral genome of the NCBI reference strain and 94–95% nucleotide sequence identity with each other.

### Animal inoculation study

The Indiana Outbreak in 2005 resulted in nearly 50% mortality of rabbits on the affected premises before intervention by the USDA. To characterize pathogenicity of the IN-05 RHDV isolate, three adult rabbits were inoculated by intramuscular injection with 1 ml each of a 10% w/v liver homogenate obtained from the index case for the IN-05 outbreak. Clinical signs of high fever and depression appeared in one animal 24 hr post inoculation. Slight nasal bleeding in two animals was apparent at 36 hr post infection, and the two symptomatic animals succumbed to the infection within 48 hr. The surviving rabbit displayed no clinical signs for three weeks post-inoculation.

**Figure 1:**
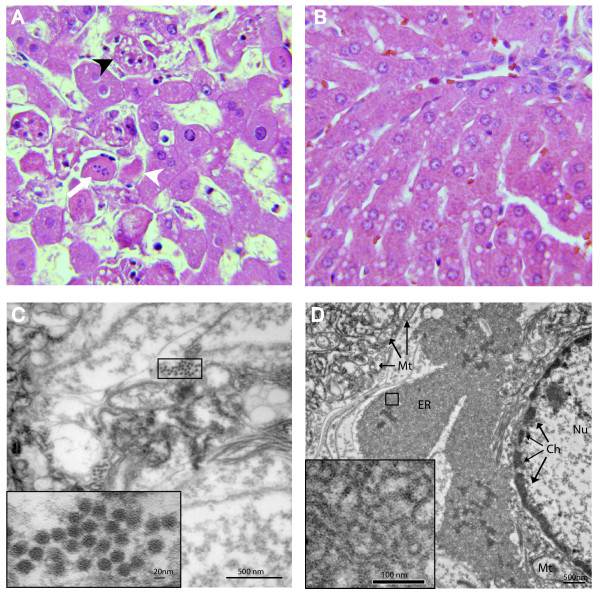
**Histopathology and cytopathology associated with IN-05 RHDVa infection**. **A**. A liver section from one of the fatally infected rabbits (day 2 post infection) is shown (H&E stain, 40× objective). Note the acute hepatocellular necrosis characterized by destruction and disassociation of hepatocytes, loss of cellular organization, and evidence of acidophilic bodies (white arrow head), karyorrhexis (white arrow), and necrotic or apoptotic hepatocytes (black arrow head). **B**. A liver section from the surviving infected rabbit (day 21 post infection) exhibited normal liver morphology (H&E staine, 40× objective). **C**. Transmission electron micrograph showing the ultrastructure of a hepatocyte from a fatally infected rabbit revealed the presence of 26.5 nm +/- 1.9 diameter viral particles with morphology characteristic of caliciviruses. **D**. An example of ultrastructural changes to a hepatocyte from one of the fatally infected rabbits. Note the margination of chromatin (Ch) in the nucleus (Nu), and disruption of cristae in mitochondria (Mt). Often, an abnormal condensation of the endoplasmic reticulum (ER) was observed. The inset shows an abnormally dense reticular network.

RHDV is known to replicate in the liver, which results in severe liver necrosis and terminally disseminated intravascular coagulation [7,43-45]. Homogenates prepared from livers of the two fatally infected animals tested positive for VP60 capsid antigen, while the liver homogenate from the surviving animal, taken on day 21 post infection, was negative for viral capsid antigen by antigen capture ELISA (Table 1). Animals that succumb to RHD typically display splenomegaly, a pale necrotic liver, and a multitude of infarcts and hemorrhages throughout the lungs. Upon necropsy, livers of the two rabbits that had died during the study were pale, and on histopathology displayed multifocal to coalescing acute severe hepatic necrosis (Figure 1A). The distribution of necrosis was mostly periportal extending towards the midzonal areas. Necrotic areas were characterized by disassociation of the hepatic cords, cellular swelling, hypereosinophilia and hepatocellular vacuolar changes (Figure 1A). Hepatocellular changes were characterized by pyknosis, karyorrhexis and karyolysis. Some of the degenerating hepatocytes contained intracytoplasmic acidophilic bodies. Infiltration by inflammatory cells was minimal and consisted mainly of neutrophils. In contrast, liver tissue from the surviving rabbit showed no evidence of necrosis or hemorrhage (Figure 1B). Lungs from the fatally infected rabbits showed pulmonary congestion and hemorrhage, and spleens were characterized by diffuse splenic congestion and mild lymphoid hyperplasia with lymphocytic apoptosis.

**Table 1:** VP60 ELISA detection in infected animals.

Sample	Surviving Rabbit	Deceased Rabbits
		
	21 dpi	2 dpi
		
Liver	-	+/+

Viral particles with short cup-like projections and a mean diameter of 26.5 +/- 1.9 nm, typical of caliciviruses [26], were evident by transmission electron microscopy of ultra-thin liver sections from the two affected animals (Figure 1C) and by negative staining electron microscopy of liver homogenates (data not shown). Cytopathic effects in hepatocytes included condensation of chromatin, and a disruption of cristae in mitochondria (Figure 1D). Many cells displayed a dense labyrinth of membrane consistent with a condensation of smooth and rough endoplasmic reticulum (Figure 1D).

Pre-inoculation serum and heparinized blood samples for all three animals were found to be negative for antibody against RHDV by ELISA (Table 2). Both serum and heparinized blood samples from the surviving rabbit tested negative until day nine post-inoculation at which time all samples tested positive for anti-VP60 IgM, IgG, and IgA until euthanasia at three weeks post-inoculation (Table 2).

**Table 2:** Serology of RHDV in experimentally infected animals.

AbELISA	Surviving Rabbit							Deceased Rabbits		
		
	0 dpi	1 dpi	2 dpi	3 dpi	9 dpi	15 dpi	21 dpi	0 dpi	1 dpi	2 dpi
		
IgM	-	nd	-	-	1:640	1:640	nd	-	nd	-
IgG	-	nd	-	-	1:40	1:40	nd	-	nd	-
IgA	-	nd	-	-	1:640	1:640	nd	-	nd	-

The diagnostic RT-PCR assay employed in all of the U.S. RHD outbreaks involved primers (88U and 315D) directed against a 246 bp region of the highly conserved RNA-dependent RNA polymerase gene (4588–4833, Materials and Methods Section). Livers from the two fatally infected animals contained RHDV genomic RNA as demonstrated by RT-PCR analysis (Table 3). Spleen and lung tissues, however, did not yield RT-PCR products nor did any of the tissue samples from the surviving animal (Table 3). Virus shedding was not detectable by RT-PCR of nasal, urethral, or rectal swabs in the fatally infected animals (Table 3). In contrast, nasal and urinary tract swabs taken at 48 hr post-inoculation and a rectal swab taken at 72 hr post-inoculation from the surviving rabbit yielded positive RT-PCR products (Table 3). While this confirmed the existence of a brief period of virus shedding during the acute phase of infection, the absence of detection in most of the swab samples suggests that detection of a carrier state or virus shedding using this RT-PCR method was not practical. Likewise, serum and heparinized blood samples from all animals were negative by RT-PCR (Table 3). While these data indicate that liver tissue represents the best sample for RHD diagnosis by RT-PCR, other more sensitive methods using either a nested RT-PCR [46] or a realtime RT-PCR [47] may be used to detect RHDV in other tissues types, blood or even paraffin embedded tissue sections.

**Table 3:** PCR detection of RHDV in experimentally infected animals.

Samples	Surviving Rabbit							Deceased Rabbits		
		
	0 dpi	1 dpi	2 dpi	3 dpi	9 dpi	15 dpi	21 dpi	0 dpi	1 dpi	2 dpi
		
Nasal Swab	-	-	+	-	-	-	-	-/-	-/-	-/-
Urethral Swab	-	-	+	-	-	-	-	-/-	-/-	-/-
Rectal Swab	-	-	-	+	-	-	-	-/-	-/-	-/-
Hep Blood	-	-	-	-	-	-	-	-/-	-/-	-/-
Plasma	-	-	-	-	-	-	-	-/-	-/-	-/-
Liver	nd	nd	nd	nd	nd	nd	-	nd	nd	+/+
Spleen	nd	nd	nd	nd	nd	nd	-	nd	nd	-/-
Lung	nd	nd	nd	nd	nd	nd	-	nd	nd	-/-

### Sequence Analysis

**Figure 2:**
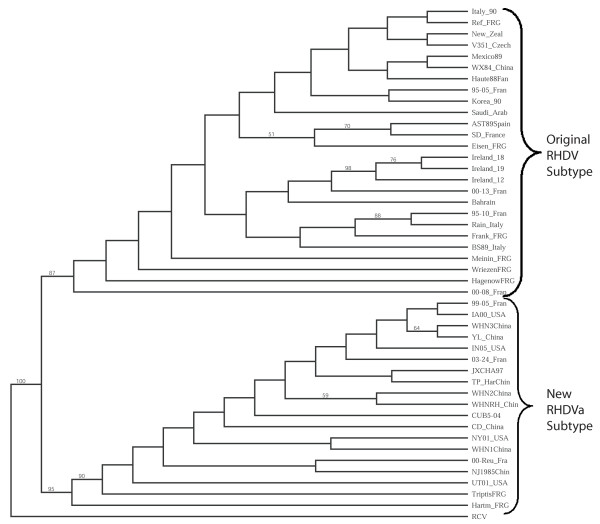
**Relationship of VP60 capsid proteins among diverse isolates of RHDV**. The predicted amino acid sequences of 45 RHDV isolates were aligned in CLUSTAL W. One thousand bootstrap replicates were subjected to protein distance and UPGMA methods and the consensus phylogenetic tree is shown. The VP60 region of a non-pathogenic rabbit calicivirus (RCV) was used as an outgroup. Two clades, one representing the original RHDV serotype and a second representing the new RHDVa subtype were identified. Bootstrap values greater than 50% are displayed above the tree branches.

**Figure 3:**
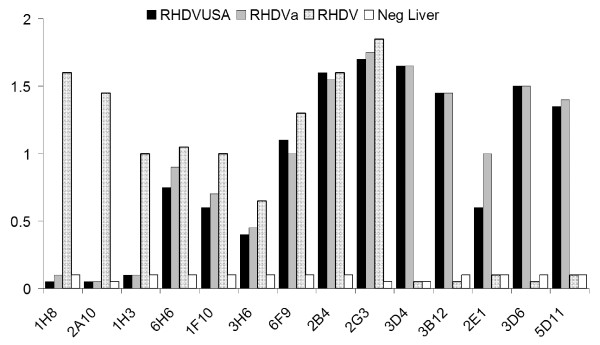
**Epitope profile of the first U.S. outbreak isolate RHDV IA-00**. The RHDV IA-00 isolate was subtyped by antigen capture ELISA using a panel of monoclonal antibodies. Previous studies and communication from Lorenzo Capucci [35] have determined that monoclonal antibodies 1H8, 2A10, and 1H3 recognize the original serotype of RHDV while antibodies 3D4, 3B12, 2E1, 3D6, and 5D11 recognize RHDVa-specific epitopes. Additional monoclonal antibodies used (6H6, 1F10, 3H6, 6F9, 2B4, and 2G3) were not subtype-specific. The IA-00 isolate (black bars) correlated in antibody recognition profile to a prototype RHDVa strain, Pavia 1997 (grey bars). The Brescia 1989 strain (stippled bars) was used as an original RHDV serotype virus control. Normal liver from an uninfected rabbit served as a negative control (white bars).

To determine whether the U.S. isolates were related to the pandemic RHDVa subtype currently spreading throughout Europe, we compared putative translations of the VP60 capsid regions for all US isolates (IA-00, NY-01, UT-01, and IN-05) to that of 41 other isolates of RHDV and RCV. All four U.S. isolates branched consistently with a group of 15 other isolates that included the typed RHDVa antigenic variants from France and Italy (Figure 2). Of note, RHDV isolates from New York and Utah in the same year, while both grouping within the RHDVa clade, did not branch together indicating separate origins for these outbreaks. Furthermore, another North American isolate, Mex-89, failed to cluster with the RHDVa clade distinguishing it from the other American isolates (Figure 2). Also consistent with the finding that this clade represented RHDVa subtypes, the IA-00 isolate was typed as RHDVa using an antigen capture ELISA and a panel of type-specific monoclonal antibodies (Figure 3). This further supports the inference that monoclonal antibody 3B12 recognizes an RHDVa type-specific epitope while monoclonal antibody 1H8 recognizes an original RHDV type-specific epitope (Figure 3) [34,35]. In contrast monoclonal antibody 2B4 recognizes a shared epitope between the two types of RHDV (Figure 3).

**Figure 4:**
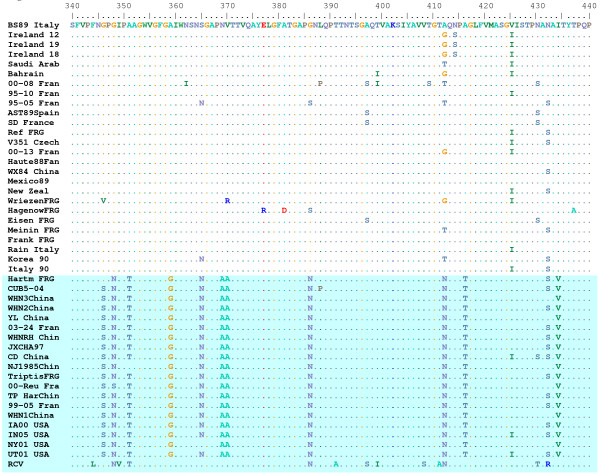
**RHDVa-specific epitope between residues 340 and 440 of the VP60 capsid protein**. A portion of the CLUSTAL W alignment of the VP60 sequence for 45 isolates of RHDV and 1 isolate of a non-pathogenic rabbit calicivirus (RCV) is shown. The top reference sequence for the alignment came from the Brescia 1989 strain (BS89 Italy) and identical amino acids were indicated by a dot. Note the large number of shared amino acid substitutions within the RHDVa clade (shaded blue).

**Figure 5:**
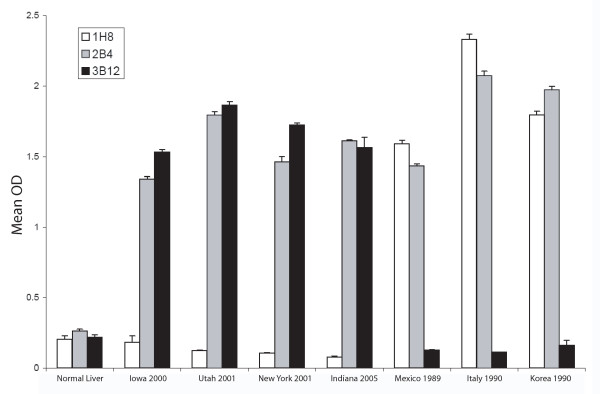
**Type-specific antigenicity of the U.S. isolates of RHDV**. Liver homogenates from experimentally infected animals were tested by antigen-capture ELISA using type-specific HRP-conjugated monoclonal antibodies (MAb). MAb 1H8 is specific for the original RHDV serotype, MAb 3B12 is specific for the new RHDVa pandemic strain, and MAb 2B4 recognizes a shared epitope. The four U.S. RHDV isolates, Mexico 1989 isolate, an Italian isolate, and Korean isolate were compared in comparison with a control liver homogenate derived from an uninfected rabbit (Normal Liver). All U.S. isolates were recognized by MAb 3B12 as belonging to the RHDVa pandemic strain.

An RHDVa strain-specific antigenic epitope has been previously predicted to reside within residues 344 to 370 in the hypervariable region E of the VP60 capsid protein [35]. Indeed, sequence alignment of the 45 RHDV isolates by CLUSTAL W [48] demonstrated that particular amino acid substitutions within this antigenic epitope are shared among the U.S. isolates and all other RHDVa serotypic variants (Figure 4). While the 344 aa-370 aa RHDVa-specific mutation cluster appeared to be the most significant cluster of type-specific mutations, additional small clusters of RHDVa-specific mutations did appear throughout the VP60 coding region (Additional file 1). To confirm the subtype-specific antigenicity of the remaining three U.S. RHDV isolates, liver homogenates from rabbits experimentally infected with each U.S. isolate were tested by antigen capture ELISA using the original RHDV strain-specific monoclonal antibody 1H8 and the RHDVa strain-specific monoclonal antibody 3B12 (Figure 5). Monocolonal antibody 2B4 was used as a control for the presence of virus and isolates from Italy, Mexico and Korea were tested for comparison to the original RHDV serotype (Figure 5). While all tested virus isolates reacted to the control antibody 2B4, only the U.S. isolates reacted with the RHDVa-specific antibody 3B12 (Figure 5). Likewise, all U.S. virus isolates failed to react with the original RHDV type-specific antibody 1H8. Conversely, isolates from Italy, Mexico and Korea, which fall outside of the RHDVa clade (Figure 2), failed to react with the RHDVa type-specific antibody 3B12 but reacted with the original RHDV type-specific antibody 1H8 (Figure 5).

**Figure 6:**
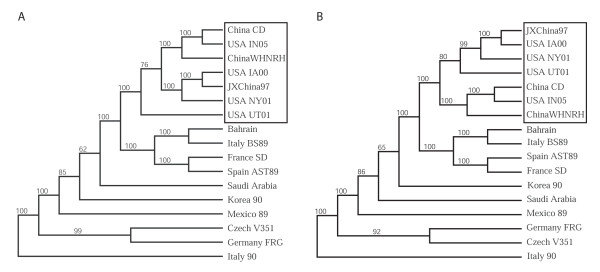
**Relationship of U.S. isolates to genomes of other RHDV isolates**. **A**. Genomes of RHDV isolates including the four U.S. isolates were aligned in CLUSTAL W and 1000 bootstrap replicates were subjected to DNA Distance and Neighbor Joining methods. A consensus tree is shown with bootstrap values greater than 50% placed above tree branches. The U.S. isolates all branched (100% of the time) with a distinct clade of RHDVa isolates from China (box). **B**. Analysis was repeated as shown in panel A. except that the VP60 coding regions were removed from the genomic sequences. All U.S. isolates continued to branch with the RHDVa isolates from China, despite removal of the RHDVa epitope.

While a more detailed look at synonymous and non-synonymous nucleotide substitutions within the VP60 coding region may be sufficient for discriminating relatedness within a single outbreak and can easily be used to discriminate between the prototype RHDV and the recent pandemic RHDVa strains, recombination or strong positive selection for particular mutations leading to RHDVa-specific epitopes could confound predictions of relatedness between geographically or temporally distant outbreaks. Therefore, to better assess the relatedness of the U.S. isolates to each other and to other geographically distinct virus isolates, we employed full genome nucleotide sequence comparisons between the 4 U.S. isolates and 10 other complete RHDV genomes (Figure 6A). Using the Neighbor Joining method and 1000 bootstrap replicates, the analysis revealed with a high degree of confidence (bootstrap values > 95%) that all 4 U.S. isolates were more closely related to separate isolates from China than they were to each other. This closer phylogenetic link between individual U.S. outbreaks and Chinese isolates indicated that each of the U.S. outbreaks were the result of a separate introduction of virus. Once again, the four U.S. isolates clustered within the RHDVa clade; therefore, to confirm our conclusions, all genomes were reanalyzed after deletion of the VP60 coding region, thus removing any potential bias attributable to recombination or positive selection for RHDVa-specific epitopes within the VP60 coding region (Figure 6B). Results were nearly identical to those obtained by using the full genomes inclusive of the VP60 coding regions confirming that the U.S. isolates were indeed more closely related to isolates from China than they were to each other (Figure 6B).

## Discussion

While the U.S. rabbit industry is clearly small as compared to other livestock industries, increased trade in global markets and the persistence and spread of RHD clearly present a risk to the U.S. domestic rabbit industry and larger rabbit industries elsewhere in the Americas. In this regard, imports of live rabbits and raw rabbit products from endemic regions present the most likely source for RHD outbreaks in the Americas. Given the enigma surrounding the sudden origins of highly pathogenic RHDV which first emerged in China in 1984 [22], and documented numerous instances of unpredictable shifts in host specificity seen in emerging pathogens from other virus families [49], it can be argued that exposure to RHDVa posses a low-probability yet potential threat to native American rabbits and other predatory animal species which may depend upon rabbits as a food source. This would require an unexpected shift in host specificity as RHDV is currently known only to cause disease in one species of rabbit, *O. cuniculus*. Such unpredictable risks however, should preclude the use of highly pathogenic viruses as biocontrol agents.

Recent recoveries of RHDV genomic RNA and subsequent phylogenetic studies on a portion of the VP60 coding region, including European rabbits predating the emergence of highly pathogenic RHDV in China, have been used to suggest that highly pathogenic RHDV may have evolved from low pathogenic RHDV independently in Europe and Asia [50-52]. These studies have focused only on a very small portion of the VP60 capsid region and genetic recombination between new and old RHDV in Europe could still explain the emergence of highly pathogenic RHDV in Europe that retains similarities to VP60 sequences of low pathogenic RHDV predating 1984. Likewise it is possible that highly pathogenic RHDV originated in Europe and rapidly diverged in Asia beginning with a very large outbreak infecting more than 200 million otherwise naive rabbits. An analysis of full genome sequences, as we have undertaken for RHDVa, needs to be undertaken in order to determine the origins of highly pathogenic RHDV. With respect to the RHDVa pandemic strain, none of the pre-1984 European isolates contain the RHDVa variant epitope suggesting that perhaps, RHDVa in Europe and elsewhere was acquired more recently from Asia.

Like the emergence of highly pathogenic RHDV, the concurrent emergence of an RHDVa subtype in Asia and Europe is quite analogous. RHDVa has been shown to be replacing the original RHDV serotype in Europe [37] and an original RHDV strain from China in 1984 (WX84 China, Figures 2 and 4) does not carry the RHDVa epitope while later isolates employed in this study do carry the RHDVa epitope (Figures 2 and 4). This fixation of RHDVa in nature is in spite of the fact that much of Europe vaccinates rabbits with a vaccine that is experimentally able to protect against both the original RHDV serotype and the new RHDVa subtype [37]. Possible explanations for this include carrier rabbits, either young rabbits which tend to be asymptomatic [3] or chronically infected rabbits that are subsequently vaccinated for RHDV. Such carriers could generate escape mutants that might later become amplified in unvaccinated animals. While a recent report shows that viral genomes persist for several months in vaccinated rabbits that have been experimentally infected with RHDV [53], persistence of infectious virus and true carrier state rabbits have yet to be demonstrated. Nevertheless, it is apparent that a selective advantage, perhaps driven by the vaccine strain being of the original RHDV serotype, is driving the fixation of the RHDVa epitope in nature.

## Conclusion

In summary, the USDA has identified four isolated outbreaks of RHD in the USA and determined complete genome sequences for the viruses responsible. The most recent of these occurred in June of 2005 in the state of Indiana. Other outbreaks in the Americas include Mexico in 1988 and more recently, in 2004, Uruguay and Cuba. As with other RHDV isolates in Europe and Asia, the Indiana RHDV isolate was found to be highly pathogenic resulting in hepatocellular necrosis, disseminated intravascular coagulation, and death. By comparative genomics we find that the USA isolates have separate origins, are most closely related to isolates from China, and that they belong to a pandemic antigenic variant strain known as RHDVa that is currently spreading throughout Europe despite implementation of an effective vaccine. This represents the first whole genome analysis and characterization of the RHDVa subtype. A close monitoring of RHDV subtype differentiation and strengthening of efforts to control the RHDVa pandemic should be undertaken to forestall the evolution of a new serotype.

## Methods

### Animal Inoculation Study

Three SPF New Zealand white rabbits, free of RHDV reactive antibodies, (Millbrook Breeding Farms) were inoculated by intra-muscular injection with 1 ml of homogenate, consisting of 10% w/v liver in 1 × PBS pH 6.4, derived from the index case of the 2005 Indiana RHD outbreak. Body temperature, heparinarinized blood, serum, nasal swabs, urinary tract swabs, and rectal swabs were taken prior to inoculation and subsequently every 24 hr during the course of infection. Two animals succumbed to the infection within 48 hr while the third fully recovered. Upon necropsy, spleen, lung, heart, and liver samples were collected for histopathology, transmission electron microscopy, RT-PCR, and antigen ELISA. Nasal swabs, urinary tract swabs, rectal swabs, and heparinized blood samples were collected for RT-PCR testing, and sera were collected for AbELISA testing. Antibody and antigen ELISA kits (OIE reference laboratory, Istituto Zooprofilattico Sperimentale della Lombardia ed Emilia Romagna, Brescia, Italy) were used to assay serum and 10% liver homogenates, respectively. Antigenic epitope analyses were performed using RHDV and RHDVa subtype-specific HRP-conjugated monoclonal antibodies [34,35] provided by Dr. Lorenzo Capucci (OIE reference laboratory) and assayed on dilutions of 10% liver homogenates using the RHDV antigen ELISA kit described above.

### Histopathology

Tissues were fixed in 10% neutral-buffered formalin, embedded in paraffin, sectioned at 5 μm thickness, stained with hematoxylin and eosin (H&E) stain, and examined by light microscopy.

### Electron Microscopy

For negative staining, liver homogenates were clarified by centrifugation at 1,500 × g for 10 min at 4°C and virus was concentrated from the supernatant by ultracentrifugation at greater than 100,000 × g and 25 psi for 30 min using a Beckman air-centrifuge. Virus pellets were re-suspended in 50 μl H_2_O applied to formvar-coated, carbon-stablized grids (Electron Microscopy Sciences) and stained with 2% phosphotungstic acid. Grids were examined with a T-7600 Hitachi electron microscope operating at 80 kV and images were recorded with a digital camera (AMT). For transmission electron microscopy, randomly-selected 2 mm × 1 cm × 1 mm pieces of rabbit liver fixed in 10% neutral buffered formalin from the two affected rabbits were re-fixed in a solution containing 2.5% glutaraldehyde in 0.1 M sodium cacodylate pH 7.4 for 24 hrs at 4°C, post fixed with 1% osmium tetroxide and 1.5% potassium ferricyanide in 0.1 M cacodylate buffer and stained *en bloc *with 2% aqueous uranyl acetate. Fixed tissues were dehydrated with an acetone series and embedded in Spurr's resin. Ultrathin sections were stained with uranyl acetate and lead citrate [54]. Images of virus and infected cells were captured as noted above and the mean diameter of 100 virus particles was determined using AMT measurement software.

### RT-PCR and Genomic Sequencing of USA and Foreign Isolates

For tissue and swab samples, total RNA was obtained using the RNeasy Mini Kit (Qiagen Inc.) and eluted in 40 μl H_2_O. For heparinized blood samples, 125 μl blood was lysed in 125 μl of H_2_O and RNA was extracted by addition of 750 μl of Trizol LS reagent (Invitrogen), precipitated in ethanol with 15 μg Glycoblue (Ambion Inc.) and resuspended in 30 μl H_2_O. In all instances, 10 μl RNA was denatured at 65°C for 10 min and set on ice for 2 min prior to cDNA synthesis at 42°C for 45 min in a 40 μl reaction using 50 ng·μl^-1 ^random hexamers (Invitrogen), 250 μM deoxynucleotides (Sigma Chemical Co.), 0.5 units·μl^-1 ^RNaseOUT (Invitrogen), 10 mM dithiothreitol, 1× First Strand Synthesis Buffer and 5 units·μl^-1 ^RT Superscript II (Invitrogen).

Diagnostic RT-PCR used for all of the U.S. RHD outbreaks was performed on 10% liver homogenates using an RT-PCR method directed against genome nucleotide positions 4588 to 4833 which represent a 246 bp portion of the RNA-dependent RNA polymerase gene. Following cDNA synthesis samples were PCR amplified using Platinum Taq Supermix (Invitrogen), 1.2 μM primer 88 U (CAAACGGAACTCACTAAAA) and 1.2 μM primer 315D (CACGCCATCATCGCCATAC). Thermocycling conditions consisted of a single denaturing step at 95°C for 9 min followed by 40 cycles of 95°C for 30 sec, 53°C for 45 sec, and 72°C for 30 sec, followed by a single 5 minute extension at 72°C. PCR products were analyzed by electrophoresis on 2% agarose E-Gels (Invitrogen). Of note, this protocol worked consistently on all tested isolates except for UT01 (data not shown).

For viral genome sequencing, alignments of representative RHDV genomes from NCBI were generated using CLUSTAL W [48] to select eight conserved primer pairs to be used in the RT-PCR of overlapping fragments of the IN-05, NY-01, UT-01, Korea-90, and Italy-90 isolates. PCR of cDNA products were then gel extracted using a QIAquick Gel Extraction Kit (Qiagen) and directly subjected to automated nucleotide sequencing on an ABI nucleotide analyzer. Sequences from the 3' end of each genome were determined by 3' RACE using an anchor primer 3'RAP: GGCCACGCGTCGACTAGTAC(T)_17 _for reverse transcription followed by PCR with the 5'3'AMP primer: GGCCACGCGTCGACTAGTAC and a conserved forward primer 3PForRHD: AGTGTTAAGATTTATAATACC. The 5' end of UT-01, NY-01, IN-05, and ITALY-90 were obtained by the 5' RACE. Random primed cDNA was tailed with dCTP and terminal deoxynucleotidyl-transferase (Invitrogen) prior to PCR with the 5'RAP primer: GGCCACGCGTCGACTAGTACGGGIIGGGIIGGGIIG and a conserved reverse primer 5pRev2RHDV: CACAAGCAGACGTTGCCGAGAT. A second round of PCR using the 5'3'AMP primer and a conserved nested reverse primer 5pRevRHDV: CCACATTTGTCACATGTCACC were used to amplify the 5' RHDV genomic ends prior to sequencing. The resulting double-strand sequence contigs were generated using CAP3 [55] to achieve genome sequences for UT-01, NY-01, IN-05, KOR 90, and ITALY 90. The complete genome of the IA-00 RHDV isolate (GenBank accession number AF258618) was determined previously. Complete genome sequences for IA-00, UT-01, NY-01, IN-05, Italy-90 and partial genomic sequence for Korea-90 were submitted to Genbank™ under the accession numbers AF258618, EU003582, EU003581, EU003578, EU003579, and EU003580, respectively.

### Phylogenetic Analyses

RHDV genomic sequences from 17 isolates were obtained by RT-PCR and direct sequencing as described above or from the NCBI database at Genbank and aligned using CLUSTAL W [48]. As some of the genome sequences lacked defined 5' or 3' termini, the 5' 74 nucleotides and 3' 56 nucleotides were trimmed from the alignment prior to the assortment of sequences using BOOTSTRAP (PHYLIP Ver. 3.66). One thousand bootstrap replicates were subjected to DNA Distance and Neighbor joining methods and a consensus phylogenetic tree was selected using the CONSENSUS algorithm (PHYLIP Ver. 3.66). DRAWTREE was used to display the results. For confirmation, the entire VP60 coding region was removed and the RHDV genome sequence analysis was repeated by an identical method.

For characterization of the VP60, the VP60 coding regions of 45 RHDV isolates and 1 RCV isolate were obtained from the NCBI database at Genbank or by direct sequencing as described above. The amino acid translations were aligned in CLUSTAL W [48] and subjected to BOOTSTRAP. One thousand bootstrap replicates were subjected to protein distance and UPGMA methods and a consensus phylogenetic tree was selected using the CONSENSUS algorithm (PHYLIP Ver. 3.66). DRAWTREE was used to display the results.

## Competing interests

The author(s) declare that they have no competing interests.

## Authors' contributions

SAM contributed in conception of the study. MTM, SCB and ZL sequenced the IN-05, UT-01, NY-01, Italy-90, and Korea-90 isolates. JGN, ZL and GW sequenced the IA-00 isolate. MTM performed the sequence analysis, FMM and TGB performed histopathology and transmission electron microscopy, and KM performed all ELISA assays. GB and LC performed the antigenic typing of IA-00. FMM led the animal inoculation study with assistance from SCB. MTM wrote the paper with contributions from all other authors. All authors read and approved the final manuscript.

## Supplementary Material

Additional file 1 Comparative analysis of the VP60 protein amino acid sequences. Comparative analysis of the VP60 protein amino acid sequences of the newly sequenced US RHDV isolates with previously elucidated sequences from GenBank. RCV is included as an out-group. The multiple-sequence alignment was compiled using the alignment tools in Bioedit. Highlighted is the highly variable E region of the VP60 capsid protein proposed to contain the conserved amino acid substitutions that characterize the RHDVa strain [35]. Click here for file
