# Supplementary material for: A pandemic strain of calicivirus threatens rabbit industries in the Americas
Source: Virol J. 2007 Oct 2;4:96. doi: 10.1186/1743-422X-4-96 (PMC2147015; doi:10.1186/1743-422X-4-96)
Supplement: Additional file 1 — Comparative analysis of the VP60 protein amino acid sequences. Comparative analysis of the VP60 protein amino acid sequences of the newly sequenced US RHDV isolates with previously elucidated sequences from GenBank. RCV is included as an out-group. The multiple-sequence alignment was compiled using the alignment tools in Bioedit. Highlighted is the highly variable E region of the VP60 capsid protein proposed to contain the conserved amino acid substitutions that characterize the RHDVa strain [35]. [file 1743-422X-4-96-S1.pdf]

```
                     10        20        30        40        50        60        70        80        90       100
             ....|....|....|....|....|....|....|....|....|....|....|....|....|....|....|....|....|....|....|....|
BS89 Italy   MEGKARTAPQGEAAGTATTASVPGTTTDGLDPGVVATTSVVTAENSSASIATAGIGGPPQQVDQQETWRTNFYYNDVFTWSVADAPGSILYTVQHSPQNN
Ireland 12   ....................................................................................................
Ireland 19   ....................................................................................................
Ireland 18   ....................................................................................................
Saudi Arab   .............................M..........I...........................................................
Bahrain      ....................................................................................................
00-08 Fran   .............................M......................................................................
95-10 Fran   ....................................................................................................
95-05 Fran   .............................M......................................................................
AST89Spain   .............................M......................................................................
SD France    .............................M......................................................................
Ref FRG      ......A......................M..........I...........................................................
V351 Czech   ......A.......R..............M..........I...........................................................
00-13 Fran   ..........SD.................M............T.........................................................
Haute88Fan   ......A......................M..........I...........................................................
WX84 China   ......A......................M..........I...........................................................
Mexico89     ......A......................M..........I...........................................................
New Zeal     ......A.........V............M..........I...........................................................
WriezenFRG   ...................................................................................VG...............
HagenowFRG   ..........D.........................................................................................
Eisen FRG    ..........AG.................M......................................................................
Meinin FRG   ...........R.................M......................................................................
Frank FRG    ....................................................................................................
Rain Italy   ....................................................................................................
Korea 90     ......A......................M..........I...........................................................
Italy 90     ......A......................M..........T...........................................................
Hartm FRG    .............................M......................................................R...............
CUB5-04      ......................L......M......A............V..................................................
WHN3China    .............................M......A............V..................................................
WHN2China    .............................M......A............V..................................................
YL China     .............................M......A............V..................................................
03-24 Fran   .............................M......A............V..................................................
WHNRH Chin   .............................M......A............V..................................................
JXCHA97      ....T........................M......A............V..................................................
CD China     .............................M......A............V..................................................
NJ1985Chin   .............................M......A............V..................................................
TriptisFRG   .............................M......A...A........V..........................................C.......
00-Reu Fra   .............................M......A............V..................................................
TP HarChin   ....T........................M......A............V..................................................
99-05 Fran   .............................M......A............V..................................................
WHN1China    .............................M......A............V..................................................
IA00 USA     .............................M......A............V..................................................
IN05 USA     .............................M......A............V..................................................
NY01 USA     .............................M......A............V..................................................
UT01 USA     .............................M......A............V..................................................
RCV          ......IT.....................M............T..A.T.V..................................................

                    110       120       130       140       150       160       170       180       190       200
             ....|....|....|....|....|....|....|....|....|....|....|....|....|....|....|....|....|....|....|....|
BS89 Italy   PFTAVLSQMYAGWAGGMQFRFIVAGSGVFGGRLVAAVIPPGIEIGPGLEVRQFPHVVIDARSLEPVTITMPDLRPNMYHPTGDPGLVPTLVLSVYNNLIN
Ireland 12   ....................................................................................................
Ireland 19   ....................................................................................................
Ireland 18   ....................................................................................................
Saudi Arab   ....................................................................................................
Bahrain      ....................................................................................................
00-08 Fran   ....................................................................................................
95-10 Fran   ....................................................................................................
95-05 Fran   ....................................................................................................
AST89Spain   ....................................................................................................
SD France    ....................................................................................................
Ref FRG      ..................................R.................................................................
V351 Czech   ..................................R.................................................................
00-13 Fran   ....................................................................................................
Haute88Fan   ....................................................................................................
WX84 China   ....................................................................................................
Mexico89     ....................................................................................................
New Zeal     ....................................................................................................
WriezenFRG   ....................................................................................................
HagenowFRG   ..................................V.................................................................
Eisen FRG    ....................................................................................................
Meinin FRG   ............................................................S.......................................
Frank FRG    ...................................................................N................................
Rain Italy   ....................................................................................................
Korea 90     ....................................................................................................
Italy 90     ....................................................................................................
Hartm FRG    ....................................................................................................
CUB5-04      ....................................................................................................
WHN3China    ....................................................................................................
WHN2China    ....................................................................................................
YL China     ....................................................................................................
03-24 Fran   ....................................................................................................
WHNRH Chin   .........................................................................................P..........
JXCHA97      ....................................................................................................
CD China     ....................................................................................................
NJ1985Chin   ....................................................................................................
TriptisFRG   ....................................................................................................
00-Reu Fra   ....................................................................................................
TP HarChin   ....................................................................................................
99-05 Fran   ....................................................................................................
WHN1China    ....................................................................................................
IA00 USA     ....................................................................................................
IN05 USA     ....................................................................................................
NY01 USA     ....................................................................................................
UT01 USA     ..........................................Q.........................................................
RCV          ....................................................................................................
```

```
                   210       220       230       240       250       260       270       280       290       300
            ....|....|....|....|....|....|....|....|....|....|....|....|....|....|....|....|....|....|....|....|
BS89 Italy  PFGGSTSAIQVTVETRPSEDFEFVMIRAPSSKTVDSISPAGLLTTPVLTGVGNDNRWNGQIVGLQPVPGGFSTCNRHWNLNGSTYGWSSPRFADIDHRRG
Ireland 12  ..........................................................................................S.
Ireland 19  ..........................................................................................S.
Ireland 18  ..........................................................................................S.
Saudi Arab  ....................................................................................................
Bahrain     ................................................A.................A.................................
00-08 Fran  ................................................A................................................Q..
95-10 Fran  ....................................................................................................
95-05 Fran  ....................................................................................................
AST89Spain  ....................................................................................................
SD France   ....................................................................................................
Ref FRG     ............................................................................................G.......
V351 Czech  ..............................................D...................................................K.
00-13 Fran  .........................V........................................................................S.
Haute88Fan  ....................................................................................................
WX84 China  ....................................................................................................
Mexico89    ....................................................................................................
New Zeal    ..................................................................................................K.
WriezenFRG  ............................................N........................V............................S.
HagenowFRG  ....C...........................A...................................................................
Eisen FRG   ...........................T.........Y..............................................................
Meinin FRG  ..........................................................V.......................................S.
Frank FRG   ....................................................................................................
Rain Italy  ....................................................................................................
Korea 90    ....................................................................................................
Italy 90    ....................................................................................................
Hartm FRG   ....T...........................A...........................................................G.......
CUB5-04     ......N...........D..........................................M......................................
WHN3China   ......N...........D.................................................................................
WHN2China   ......N...........D.................................................................................
YL China    ......N...........D.................................................................................
03-24 Fran  ......N...........D.................................................................................
WHNRH Chin  ......N...........D.................................................................................
JXCHA97     ......N...........D.................................................................................
CD China    ......N...........D.................................................................................
NJ1985Chin  ......N...........D.................................................................................
TriptisFRG  ......................................................................VF............................
00-Reu Fra  ......N...........D.................................................................................
TP HarChin  ......N...........D.................................................................................
99-05 Fran  ......N...........D.................................................................................
WHN1China   ......N...........D.................................................................................
IA00 USA    ......N...........D.................................................................................
IN05 USA    ......N...........D.................................................................................
NY01 USA    ......N.............................................................................................
UT01 USA    ....................................................................................................
RCV         ....................................VT............T.....C...........L.....................T......A

                   310       320       330       340       350       360       370       380       390       400
            ....|....|....|....|....|....|....|....|....|....|....|....|....|....|....|....|....|....|....|....|
BS89 Italy  SASYPGSNATNVLQFWYANAGSAVDNPISQVAPDGFPDMSFVPFNGPGIPAAGWVGFGAIWNSNSGAPNVTTVQAYELGFATGAPGNLQPTTNTSGAQTV
Ireland 12  ....................................................................................................
Ireland 19  ....................................................................................................
Ireland 18  ....................................................................................................
Saudi Arab  ......G................I............................................................................
Bahrain     ..................................................................................................I.
00-08 Fran  ....N.G................I.....................................I.........................P........S.I.
95-10 Fran  ......N.............................................................................................
95-05 Fran  ........P..............I........................................N....................S..............
AST89Spain  ......N................I........................................................................S...
SD France   ......N................I........................................................................S...
Ref FRG     ....S..................I............................................................................
V351 Czech  .......................I...........L................................................................
00-13 Fran  ......N.............................................................................................
Haute88Fan  ....S.N................I............................................................................
WX84 China  .......................I............................................................................
Mexico89    .......................I............................................................................
New Zeal    .......................I............................................................................
WriezenFRG  .......................I.....................V.......................R..............................
HagenowFRG  .......................I.....................................................R...D....S..............
Eisen FRG   .......................I........................................................................S...
Meinin FRG  ......N.............................................................................................
Frank FRG   ......N.............................................................................................
Rain Italy  ......N.............................................................................................
Korea 90    ....S..................I........................................N...................................
Italy 90    ..............S........I............................................................................
Hartm FRG   ......N.S..............I.......................N..T.......G.....N...AA...............N..............
CUB5-04     ....S.N.S..............I.....................S.N..T.......G.....N...AA...............N.P............
WHN3China   ....S.N.S..............I.....................S.N..T.......G.....N...AA...............N..............
WHN2China   ....S.N.S..............I.....................S.N..T.......G.....N...AA...............N..............
YL China    ....S.N.S..............I.....................S.N..T.......G.....N...AA...............N..............
03-24 Fran  ....S.N.S..............I.....................S.N..T.......G.....N...AA...............N..............
WHNRH Chin  ....S.N.S..............I.....................S.N..T.......G.....N...AA...............N..............
JXCHA97     ....S.NSS..............I.....................S.N..T.......G.....N...AA...............N..............
CD China    ....S.N.S..............I.....................S.N..T.......G.....N...AA...............N..............
NJ1985Chin  ....S.N................I.....K...............S.N..........G.....N...AA...............N..............
TriptisFRG  ....S.N.S..............I.....................S.N..T.......G.....N...AA...............N..............
00-Reu Fra  ....S...S..............I.....................S.S..T.......G.....N...AA...............N..............
TP HarChin  ....S.N.S..............I.....................S.N..T.......G.....N...AA...............N..............
99-05 Fran  ....S.N.S..............I.....................S.N..T.......G.....N...AA...............N..............
WHN1China   ...FA.N.S..............I.....................S.N..T.......G.....N...AA...............N..............
IA00 USA    ....S.N.S..............I.....................S.N..T.......G.....N...AA...............N..............
IN05 USA    ....S.N.S..............I.....................S.N..T.......G.....N...AA...............N..............
NY01 USA    ...FA.N.S..............I.....................S.N..T.......G.........AA...............N..............
UT01 USA    ....S.N.S..............I.....................S.N..T.......G.....N...AA...............N..............
RCV         A..Q.A-AVA..................C..............L...NV.T..................................N....A.....S.I.
```

```
                    410       420       430       440       450       460       470       480       490       500
             ....|....|....|....|....|....|....|....|....|....|....|....|....|....|....|....|....|....|....|....|
BS89 Italy   AKSIYAVVTGTAQNPAGLFVMASGVISTPNANAITYTPQPDRIVTTPGTPAAAPVGKNTPIMFASVVRRTGDVNATAGSANGTQYGTGSQPLPVTIGLSL
Ireland 12   ...........G.S..........I................................................S...T....................
Ireland 19   ...........G.S..........I................................................A...T....................
Ireland 18   ...........G.S..........I................................................A...T....................
Saudi Arab   ...........T............I................................................S....S...................
Bahrain      ...........G............I....................................................T....................
00-08 Fran   ........S..T.................S...................................I.........S........................
95-10 Fran   ........................I................................................S....S...................
95-05 Fran   ...........T...................S....................................................................
AST89Spain   .............................S......................................................................
SD France    .............................S.........................................................R............
Ref FRG      ........................I......S....................................................................
V351 Czech   ........................I......S....................................................................
00-13 Fran   ...........G............I................................................A...T....................
Haute88Fan   ....................................................................................................
WX84 China   ...............................S....................................................................
Mexico89     ............................................................................V.......................
New Zeal     ........................I......S...............................................V....................
WriezenFRG   ...........G............I....................................................T..Y.................
HagenowFRG   ....................................A...........................................V....................
Eisen FRG    .............................S......................................................................
Meinin FRG   ...........T...................S........N...........................................................
Frank FRG    ..................................................................................S.................
Rain Italy   ........................I................................................S..........................
Korea 90     ...........T...................S............................................V.......................
Italy 90     ........................I......S....................................................................
Hartm FRG    ...........N...T...............S.V..................................................................
CUB5-04      ...........N...T...............S.V..............................P...........A...T....................
WHN3China    ...........N...T.................V..........................................A...T....................
WHN2China    ...........N...T...............S.V..........................................A.......................
YL China     ...........N...T.................V..........................................A...T....................
03-24 Fran   ...........N...T...............S.V..............................................T....................
WHNRH Chin   ...........N...T...............S.V..........................................A.......................
JXCHA97      ...........N...T...............S.V..........................................A...T....................
CD China     ...........N...T........I....S.S.V.........................................VA...TS...................
NJ1985Chin   ...........N...T.................V.......................S..................A...T....................
TriptisFRG   ...........N...T...............S.V..........................................A...T....................
00-Reu Fra   ...........N...T.................V..........................................A...T....................
TP HarChin   ...........N...T...............S.V..........................................A...T....................
99-05 Fran   ...........N...T...............S.V..........................................A...T....................
WHN1China    ...........N...T.................V.................................K........A...T....................
IA00 USA     ...........N...T...............S.V..........................................A...T....................
IN05 USA     ...........N...T........I......S.V..........................................A...T....................
NY01 USA     ...........N...T.................V..........................................A.......................
UT01 USA     ...........N...T........I......S.V..........................................A...T....................
RCV          .......S..AN.................T.R............NA............V................E...D....................

                    510       520       530       540       550       560       570       580
             ....|....|....|....|....|....|....|....|....|....|....|....|....|....|....|....|
BS89 Italy   NNYSSALMPGQFFVWQLTFASGFMEIGLSVDGYFYAGTGASTTLIDLTELIDVRPVGPRPSKSTLVFNLGGTANGFSYV*
Ireland 12   .......................................................................A.......*
Ireland 19   .......................................................................A.......*
Ireland 18   .......................................................................A.......*
Saudi Arab   .......................................................................A.......*
Bahrain      ...............................................................................*
00-08 Fran   .......................................................................A.......*
95-10 Fran   ........................................L......................................*
95-05 Fran   ...............................................................................*
AST89Spain   ...............................................................................*
SD France    ...............................................I...............................*
Ref FRG      ...............................................................................*
V351 Czech   ...............................................................................*
00-13 Fran   .......................................................................A.......*
Haute88Fan   ...............................................................................*
WX84 China   ...............................................................................*
Mexico89     ...............................................................................*
New Zeal     ...............................................................................*
WriezenFRG   ......................................................................SA.......*
HagenowFRG   ...............................................A...............................*
Eisen FRG    ...............................................................................*
Meinin FRG   ...................................................................S....T......*
Frank FRG    .....S..................................L......................................*
Rain Italy   ........................................L......................................*
Korea 90     ...............................................................................*
Italy 90     ...............................................................................*
Hartm FRG    .......L...............................................................V.......*
CUB5-04      .......I................................................................T......*
WHN3China    ........................................................................T......*
WHN2China    .......V................................................................T......*
YL China     ........................................................................T......*
03-24 Fran   ........................................................................T......*
WHNRH Chin   .......V................................................................T..L...*
JXCHA97      ........................................................................T......*
CD China     .......................................................................AT......*
NJ1985Chin   ...............................................................................*
TriptisFRG   .......V.......................................................................*
00-Reu Fra   .......................................S.......................................*
TP HarChin   ..H.....................................................................T......*
99-05 Fran   ........................................................................T......*
WHN1China    .......................................................................AT......*
IA00 USA     ........................................................................T......*
IN05 USA     ........................................................................T......*
NY01 USA     ........................................................................T......*
UT01 USA     ...............................................................................*
RCV          .......T.........N..........N.......................I........T.........ATS.....*
```
